# High number of CD56^bright^ NK-cells and persistently low CD4^+^ T-cells in a hemophiliac HIV/HCV co-infected patient without opportunistic infections

**DOI:** 10.1186/1743-422X-10-33

**Published:** 2013-01-26

**Authors:** Giulia Fregni, Anaenza Freire Maresca, Valérie Jalbert, Anne Caignard, Daniel Scott-Algara, Elisabeth Bordé Cramer, Elisabeth Rouveix, Marie C Béné, Claude Capron

**Affiliations:** 1 Inserm U1016, Institut Cochin, Département d’Hématologie et d’Immunologie, Paris, France; 2 Université Paris Descartes Sorbonne Paris Cité, CNRS (UMR8104), Paris, France; 3 Service d’Hématologie et d’Immunologie, Hôpital Ambroise Paré, Boulogne-Billancourt, France; 4 UFR des Sciences et de la Santé Paris Ile-de-France Ouest, Université de Versailles St Quentin en Yvelines, St Quentin en Yvelines, France; 5 Laboratoire d’Hématologie, Hôtel-Dieu-CHU, Nantes, France; 6 Service de Médecine Interne, Hôpital Ambroise Paré, Boulogne-Billancourt, France; 7 Unité de Régulation des Infections Rétrovirales, Institut Pasteur, Paris, France; 8 Institute of Pathology, Centre Hospitalier Universitaire Vaudois, Faculty of Biology and Medecine, University of Lausanne, Lausanne, Switzerland

**Keywords:** HIV, HCV, Opportunistic infections, CD4, NK cells

## Abstract

**Background:**

Both the human immunodeficiency virus (HIV) and hepatitis C virus (HCV), either alone or as coinfections, persist in their hosts by destroying and/or escaping immune defenses, with high morbidity as consequence. In some cases, however, a balance between infection and immunity is reached, leading to prolonged asymptomatic periods. We report a case of such an indolent co-infection, which could be explained by the development of a peculiar subset of Natural Killer (NK) cells.

**Results:**

Persistently high peripheral levels of CD56^+^ NK cells were observed in a peculiar hemophiliac HIV/HCV co-infected patient with low CD4 counts, almost undetectable HIV viral load and no opportunistic infections. Thorough analysis of NK-subsets allowed to identify a marked increase in the CD56^bright/dim^ cell ratio and low numbers of CD16^+^/CD56^-^ cells. These cells have high levels of natural cytotoxicity receptors but low NCR2 and CD69, and lack both CD57 and CD25 expression. The degranulation potential of NK-cells which correlates with target cytolysis was atypically mainly performed by CD56^bright^ NK-cells, whereas no production of interferon γ (IFN-γ) was observed following NK activation by K562 cells.

**Conclusions:**

These data suggest that the expansion and lytic capacity of the CD56^bright^ NK subset may be involved in the protection of this « rare » HIV/HCV co-infected hemophiliac A patient from opportunistic infections and virus-related cancers despite very low CD4^+^ cell counts.

## Background

Hemophiliac patients have been especially at risk of infections by blood-borne viruses before drastic measures were taken to control transfused blood products. We report the peculiar case of a hemophiliac patient co-infected by both human immunodeficiency virus (HIV) and hepatitis C virus (HCV) who developed unusual immune responses.

The course of HIV infection is in most cases quite straightforward. Both the cytopathic effect of the virus and cytotoxic immune responses contribute to the destruction of infected CD4^+^ cells. The classically reverted CD4^+^/CD8^+^ ratio is accompanied with high viral loads. In most cases, long-term anti-viral therapy results in a near disappearance of the viral load together with a progressive increase in CD4^+^ counts. However, in rare cases, the viral load may become undetectable while CD4^+^ counts remain low [1].

Approximately 15–30% of HIV patients present HCV co-infection. HCV is also the most common cause of chronic liver disease and leading cause of death in hemophiliacs [2]. Co-infection usually leads to a more rapid progression towards liver fibrosis and death compared to single HCV infection while the effect of HCV on HIV infection is still debated [3,4].

The peculiar case that we report is of a long-term clinically asymptomatic hemophiliac patient co-infected with HIV and HCV since childhood. It sheds new light on the possible immunological adaptation mechanisms liable to control HIV infection. A role of Natural Killer (NK) cells in the non-progression of both infections is likely according to the unusual NK-subsets observed in this patient [5].

## Case presentation

A 38 years-old moderate hemophiliac A male patient was diagnosed to be co-infected with HIV (Subtype B, CCR5^+^) and HCV (Subtype 4), both transmitted by a blood transfusion received in 1982 during hip surgery for a fracture. Diagnoses of these infections were respectively ascertained in 1988 and 1998.

**Figure 1:**
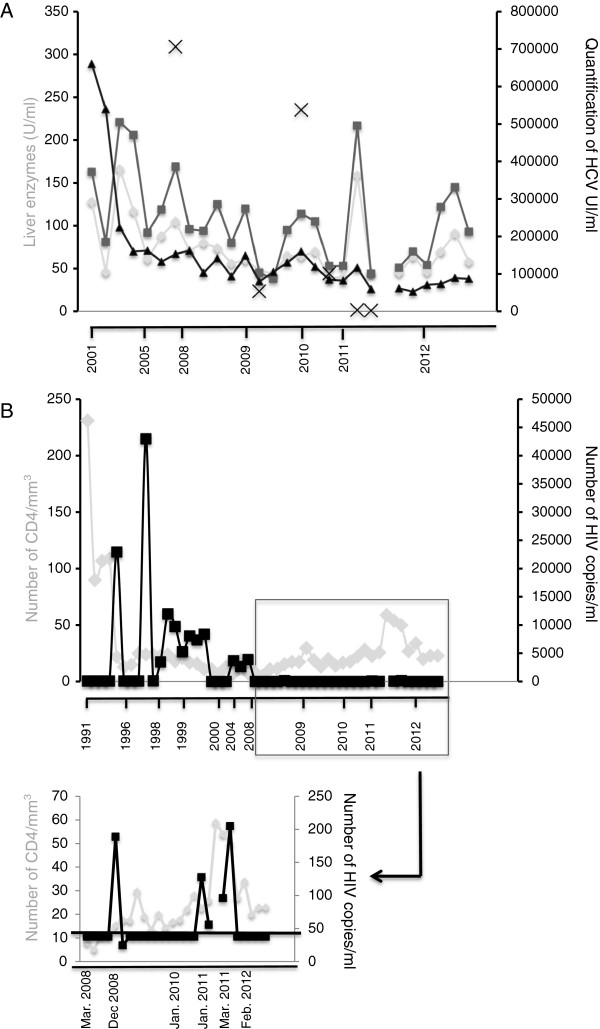
**HIV and HCV viral loads, hepatic functions and CD4 count. A**: Hepatic functions determined by liver enzyme assays (ASAT: L-aspartate aminotransferase (light grey circles) (U/L); ALAT: L-alanine aminotransferase (grey squares) (UL/) and γGT: gamma-glutamyltransferase (triangles)(U/L)) and HCV viral load (black crosses, number of HCV UI/mL) over time. **B**: Absolute numbers of CD4^+^ T-cells (grey line, number of CD4/mm3) and HIV viral load (black line, number of copies/mL; limit of detection: 40 copies/ml) over time.

Since 1989, he received different lines of anti-retroviral treatment (ART) then HAART (Highly Active Anti-Retroviral Therapy) with poor compliance, voluntary interruption until 2008, and correct compliance since then. A 215S mutation of the HIV virus reverse transcriptase, which induces resistance to the anti-retroviral drug azidothymidine was consequently identified. His HCV infection (Metavir score A0A1-F1F2 determined by biopsy and checked regularly by noninvasive markers of hepatitis inflammation/fibrosis) was never treated. Moreover his hepatic assessment, as observed with L-aspartate aminotransferase (ASAT), L-alanine aminotransferase (ALAT) and γGT gamma-glutamyltransferase markers is not so much disrupted (Figure 1A).

The patient received prosthesis of the left eye in 1996 after a paintball accident and a hip replacement in 2011 for osteo-necrosis known since 1982. None of these surgeries were accompanied by infectious side effects. Indeed, over these 30 years and despite an extremely low number of T lymphocytes, the patient displayed neither opportunistic infections, nor HIV-related cancer, nor HCV-hepatitis damages. In 1989 his initial CD4^+^ T-cells count was 196/mm^3^ and since 1995 remains between 5 and 59/mm^3^ (Figure 1B). HIV viral loads have been mostly undetectable (below 40 copies/mL) or very low (matching deliberate HAART interruptions) since 2008 (Figure 1B).

**Figure 2:**
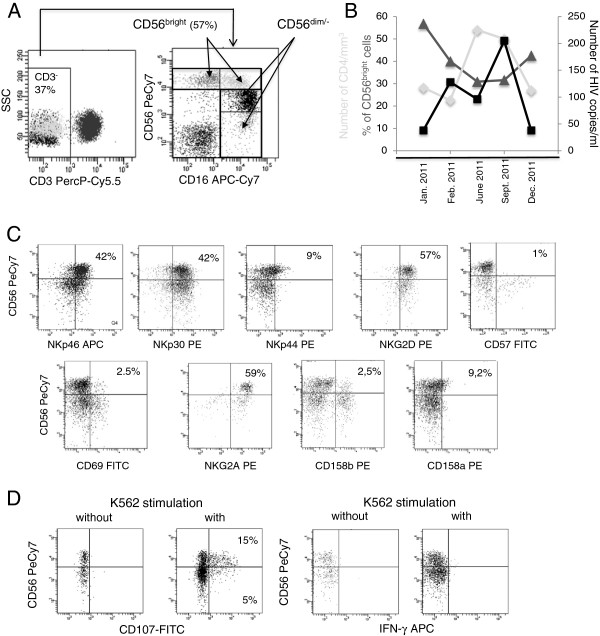
**Percentages of Natural Killer (NK) cells and their subsets; lytic potential of NK cells. A**: Representative dot plot of CD16^low^CD56^bright^; CD16^+^CD56^bright^, CD16^+^, CD56^dim^ and CD16^bright^CD56^-^ cells. Based on forward and side scatter analysis, cells were first gated on lymphocytes (not shown) and then on CD3^-^ cells. **B**: Absolute numbers of CD4^+^ T-cells (light grey line, number of CD4/mm3), % of CD56^bright^ in total CD56 NK cells (dark grey line, % of CD56^bright^ cells) and HIV viral load (black line, number of copies/mL) during one year. **C**: Representative dot plots of the expression of activating and inhibitory receptors on NK-cells among CD56^+^ CD16^+/−^ cells. **D**: Representative dot plots of CD107a degranulation and IFN-γ secretion on CD56^dim^ and CD56^bright^ cells, following PBMC activation by K562.

Over the past two years, the immunophenotypic and functional status [6] of his CD3^-^CD56^+^ NK-cells were explored by multi-parameter flow cytometry (reagents from Becton Dickinson, MountainView, CA, Figures 2A-D). Surprisingly, an abnormally high percentage of NK-cells has been steadily observed, representing approximatively 40% of peripheral lymphocytes and characterized by an expansion of the CD56^bright^ subset (~30 to 50% of NK-cells) (Figures 2A and 2B). The percentage of CD56bright cells seems to increase when the number of CD4 decreases moreover HIV viral load is below 40 copies/ml when % of CD56bright cells is the more important (Figure 2B). These cells display high levels of natural cytotoxicity receptors NCR1 (NKp46), NCR3 (NKp30) and NKG2D compared to CD56^dim^ cells, and have a low expression of NCR2 (NKp44) and CD69, with no labeling for CD57 nor CD25 (data not shown). The inhibitory CD94/NKG2A receptor is expressed by all CD56^bright^ cells and a majority of CD56^dim^ NK-cells while there is a low expression of CD158b on CD56^dim^ and CD56^bright^ NK-cells and a faint labeling with CD158a (Figure 2C).

Surprisingly, the NK degranulation potential (% of CD107a) that correlates with target cytolysis [7,8], usually exerted by the CD56^dim^ NK subset [9], is here mainly performed by CD56^bright^ NK-cells. Strikingly, these cells did not produce IFN-γ following NK activation by K562 (Figure 2D).

In addition, no Th1/Th2 cytokines (TNF-α, IFN-γ, IL-2, IL-4, IL-5, IL-10) have been detected in the patient’s serum (data not shown).

## Discussion

Several studies have reported that in HIV infection, and even more in HIV/HCV co-infection, NK cells most often lack CD56 expression and have impaired functions [10-12], opposite to what was observed in the patient reported here. However, at least one study [13] reported a similar quantitative imbalance of CD56^bright^ and CD56^dim^ NK-cells in HIV or HCV patients as shown here. Two possibilities could explain this profile. The first is an alteration in the tissue localization of NK-subsets in tissues as there could be selective trapping in the liver in HCV infection or in lymph nodes (LN) in HIV infection [13]. Secondary lymphoid tissues, and particularly LN, are important sites for HIV replication and NK development. In HIV patients, the disruption of LN architecture could interfere with the numbers of CD56^bright^ cells reaching the circulation [14], and the increase of circulating CD56^bright^ NKG2A^+^ cells could thus be caused by NK activation and release from LN [15,16].

The other hypothesis is that impairment in NK-cell homeostasis, in HIV and HCV infections, could result from different behaviors of NK-subsets. An imbalance between CD56^+^ NK-subsets could indeed result from an enhanced production, expansion or survival of the CD56^bright^ pool accompanied by a decreased generation or survival of CD56^dim^ NK-cells [17].

When altered CD56^dim/bright^ NK-cell ratios have been reported in asymptomatic HIV or HCV patients with low CD4^+^ counts, viral loads remained detectable [10,13]. Ironson et al. [16] indeed described a rare group of HIV patients with very low CD4^+^ T-cells counts who displayed prolonged asymptomatic periods. These patients presented higher NK-cell numbers and cytotoxic potential than HIV patients with high CD4^+^ counts. However, unlike the patient we report upon, they retained an elevated viral load [16].

The particular immunophenotype observed here, characterized by high CD56^bright^ numbers, recalls that of NK-cells from low viremic HIV patients with high numbers of CD4^+^ T-cells [14], yet our patient never recovered normal CD4^+^ counts.

The cytokine microenvironment may also alter the immunophenotype of NK-cells. Since IL15 and/or IL21 [13,18] regulate NK homeostasis and effector functions, the absence of IL-15 production could favor CD56^bright^ NK-cells and thus induce a change in CD56^bright/dim^ proportions. Indeed, no IL-15 could be detected in the patient’s serum.

NKp44, usually induced on activated NK-cells, has been shown to be overexpressed by HIV patients with low CD4^+^ T-cells [19] and to be involved in CD4^+^ T-cell lysis [20,21]. However, in the present case, NKp44 is faintly expressed by CD56^bright^ NK-cells, thus likely not involved in CD4^+^ depletion through this pathway. High NKG2A expression, as observed here, has conversely been associated with the escape of HIV infected CD4^+^ T-cells from NK-cell killing [22]. We cannot however exclude a lysis of CD4 cells by NK-cells, since the patient’s CD56^bright^ cells were clearly efficiently activated by the K562 cell line [7,8].

The replacement of CD56^+^ NK-cells by functionally defective CD56^-^ NK-cells is one of the mechanisms underlying the impaired NK response commonly observed in HIV and HIV/HCV patients [23,24]. However, in the present case, CD56^-^CD16^+^ cell numbers are low and CD56^bright^ cells are functional, suggesting that an unusual scenario is involved to contain HIV and possibly HCV [5] infection.

As a whole, the functional status of this patient’s NK-cells is different from that observed in HIV or HCV infected patients for whom NK cytotoxicity relies on the CD56^dim^ compartment [10,13].

It is finally of interest to note that hemophiliac patients with HCV or HCV/HIV infections have been shown to be at risk of developing cancers [25]. The special NK responses observed here could therefore also be involved in the protection of this patient towards the development of secondary complications linked to HIV/HCV infections.

## Conclusions

It is well established that HCV disease progresses more rapidly in HCV/HIV-1 co-infected patients [3,26-28]. This co-infection is probably also associated with a more rapid progression of HIV disease [29,30]. Although the efficacy of anti-retroviral therapy has increased the life expectancy of HIV-infected individuals, the management of co-infected patients is still a challenging field. Indeed, co-infection complicates the treatment of both infections due to impaired liver function, drug toxicity, reduced responsiveness to HCV treatment with pegylated infereron and ribavirin, and poor immune restoration upon ART [31]. HCV/HIV co-infection represents a challenge for the immune system and NK-cells are probably important at all stages to manage the viral attack.

Despite very low CD4^+^ T cell counts, the special long-term HIV/HCV co-infected hemophiliac patient that we describe here is healthy and presents neither opportunistic infections nor any sign of virus-related cancer. This patient shows unexpected NK-cell populations characterized by an abnormally high proportion of the CD56^bright^ NCR^+^ NK-cell subset. These cells are also unusually endowed with cytotoxic potential and no IFN-γ production. It is highly suspected that these cells are involved in both the destruction of his CD4^+^ T-cells and viral control. Such control of the viral load is a rare case of immunological adaptation to infections that outlines the plasticity of the immune system when confronted with unusual aggressions from the environment.

The successful control established by this patient over both his HIV and HCV infections and to avoid opportunistic infections sheds new light on the compliance of the immune system together with HAART and opens new original research fields in order to strengthen control mechanisms for the management of viral infections via the enhancement of innate immunity [32].

### Ethical approval

Ethics committee, CPP (Comité de Protection des Personnes) has given ethical approbation. Informed consent has been signed by the patient. These two documents have been joined to the paper.

## Abbreviations

NK: Natural Killer; HIV: Human immunodeficiency virus; HCV: Hepatitis C virus; NCR: Natural cytotoxicity receptor; KIR: Killer-cell immunoglobulin-like receptors; Th: Lymphocyte T helper; TNFα: Tumor necrosis factor; IFNγ: Interferon; IL: Interleukin (IL2, IL4, IL5, IL10); ART: Antiretroviral treatment; LN: Lymph node.

## Competing interests

The authors declare that they have no competing interests.

## Authors’ contribution

Giulia Fregni and Valérie Jalbert performed experiments. Anaenza Freire Maresca, Anne Caignard and Marie C Béné drafted the manuscript or revised it. Daniel Scott-Algara, Elisabeth Bordé Cramer and Elisabeth Rouveix gave final approval of the version to be published. Claude Capron initiated and supervised the study, wrote and edited the manuscript. All authors read and approved the final manuscript.

## Authors’ information

Giulia Fregni is a Ph D student; Valérie Jalbert is a laboratory technician; Anne Caignard and Daniel Scott-Algara are researchers; Marie C Béné, Elisabeth Bordé Cramer and Elisabeth Rouveix are university professors; Anaenza Freire Maresca is a clinician; Claude Capron is a university assistant professor.
